# Cuproptosis: Mechanisms, biological significance, and advances in disease treatment—A systematic review

**DOI:** 10.1111/cns.70039

**Published:** 2024-09-12

**Authors:** Chengliang Pan, Zhilin Ji, Qingxuan Wang, Zhao Zhang, Zhenchuan Wang, Chen Li, Shan Lu, Pengfei Ge

**Affiliations:** ^1^ Department of Neurosurgery First Hospital of Jilin University Changchun P.R. China

**Keywords:** apoptosis, autophagy, cancer, cell death, copper, cuproptosis, tricarboxylic acid cycle

## Abstract

**Background:**

Copper is an essential trace element for biological systems, as it plays a critical role in the activity of various enzymes and metabolic processes. However, the dysregulation of copper homeostasis is closely associated with the onset and progression of numerous diseases. In recent years, copper‐induced cell death, a novel form of cellular demise, has garnered significant attention. This process is characterized by the abnormal accumulation of intracellular copper ions, leading to cellular dysfunction and eventual cell death. Copper toxicity occurs through the interaction of copper with acylated enzymes in the tricarboxylic acid (TCA) cycle. This interaction results in subsequent protein aggregation, causing proteotoxic stress and ultimately resulting in cell death. Despite the promise of these findings, the detailed mechanisms and broader implications of cuproptosis remain underexplored. Therefore, our study aimed to investigate the role of copper in cell death and autophagy, focusing on the molecular mechanisms of cuproptosis. We also aimed to discuss recent advancements in copper‐related research across various diseases and tumors, providing insights for future studies and potential therapeutic applications.

**Main Body:**

This review delves into the biological significance of copper metabolism and the molecular mechanisms underlying copper‐induced cell death. Furthermore, we discuss the role of copper toxicity in the pathogenesis of various diseases, emphasizing recent advancements in the field of oncology. Additionally, we explore the therapeutic potential of targeting copper toxicity.

**Conclusion:**

The study highlights the need for further research to explore alternative pathways of copper‐induced cell death, detailed mechanisms of cuproptosis, and biomarkers for copper poisoning. Future research should focus on exploring the molecular mechanisms of cuproptosis, developing new therapeutic strategies, and verifying their safety and efficacy in clinical trials.

## Graphical abstract

Copper is an essential trace element in the human body, participating in a variety of biological functions and metabolic processes. The homeostatic copper in the human body is regulated by the absorption, transportation, storage, and metabolism of copper. Dysregulation of copper metabolism is closely related to the occurrence and progression of various diseases. The cell death induced by copper overload is initiated by interaction between copper and acylated enzymes in the tricarboxylic acid (TCA) cycle, which then results in protein aggregation, loss of iron‐sulfur cluster proteins, and cell death. Furthermore, we discuss the role of copper in regulating autophagy, the relationship between copper‐dependent death and disease, and the potential role of copper in cancer treatment. We emphasize the necessity for further research into alternative pathways of copper‐induced cell death, the detailed mechanisms of Cuproptosis, and biomarkers for copper poisoning. It proposes future research directions, including exploring the molecular mechanisms of Cuproptosis, developing new therapeutic strategies, and verifying their safety and efficacy in clinical trials.

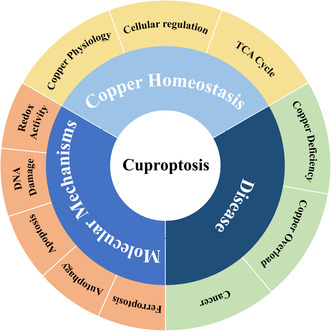

## 1 BACKGROUND

Copper, an essential trace metal, plays a crucial role in cellular physiology and metabolism. It functions as a cofactor for various enzymes and regulates cellular signal transduction and gene expression. However, the homeostasis of copper ions is vital for cellular function, as abnormal accumulation or deficiency can lead to cellular dysfunction or death. The recent discovery of cuproptosis, a novel form of copper‐dependent cell death, has attracted widespread attention.^1^ Cuproptosis involves the binding of copper ions to acylated tricarboxylic acid (TCA) cycle proteins in the mitochondria. This binding triggers protein aggregation and the loss of iron–sulfur cluster proteins, ultimately leading to cell death through a nontraditional apoptotic pathway. The discovery of cuproptosis provides a new perspective on the significance of copper in cellular metabolism and offers new strategies for treating diseases associated with copper metabolism disorders.

The regulation of copper ions has emerged as a novel therapeutic approach in the treatment of disease.^2^ Copper chelating agents have shown the potential to inhibit the proliferation of tumor cells by reducing the bioavailability of copper that is available within the cell. Conversely, copper ionophores exert an anti‐tumor effect by increasing intracellular copper levels, inducing copper overload and cytotoxicity. The anti‐tumor activity of these copper‐based compounds may be attributed to their impact on copper metabolism within the tumor microenvironment, stimulation of immune responses, and synergistic effects with other chemotherapy drugs.

Additionally, the role of copper in regulating the process of autophagy has attracted the attention of researchers.^3^ Autophagy is an intracellular process that involves the degradation and recycling of cellular components, and it plays a crucial role in maintaining intracellular homeostasis. Both an excess and deficiency of copper can affect the process of autophagy, thereby influencing the cell's response to stress and susceptibility to death. Copper regulates autophagy by affecting the AMPK‐MTOR signaling pathway, directly interacting with autophagy‐related proteins or modulating oxidative stress. Copper‐induced autophagy may help protect cells from damage, while in certain cases, copper may promote autophagy‐dependent cell death.

In summary, cuproptosis, an emerging mechanism of cell death, holds significant biological importance and demonstrates great potential in the treatment of diseases. Therefore, by reviewing the homeostasis of copper within biological systems, we aimed to gain an in‐depth understanding of the role of copper in cell death and autophagy, discuss the recent advancements in copper‐related research in various diseases and tumors, and provide insight for future studies and applications of cuproptosis.

## 2 MAIN TEXT

### 2.1 Copper homeostasis in the body

#### 2.1.1 Copper physiology in the body

Copper is an essential micronutrient in the human body, crucial for a variety of biological functions.^4^, ^5^ The absorption, transportation, storage, and metabolism of copper in the human body is a complex and meticulously regulated process. Copper is primarily absorbed in the small intestine, especially in the duodenum and jejunum.^6^ The presence of copper in the diet is primarily in the form of inorganic compounds or as part of organic substances. Copper absorption in the small intestine is predominantly carried out through an active transport mechanism that involves specific copper transport proteins such as copper transport protein 1 (CTR1) and ATP7A.^7^ These transport proteins use the energy generated from the hydrolysis of ATP within the cell to transport copper ions (Cu^2+^) from the intestinal lumen into the enterocytes. In addition, some copper can also permeate the cells through passive diffusion.^8^

After entering the enterocytes, copper binds to ceruloplasmin, a plasma protein with a high copper‐binding capacity, responsible for transporting copper throughout the body.^9^ Ceruloplasmin not only transports copper but also possesses antioxidant properties, capable of eliminating free radicals within the body.^10^ In the bloodstream, copper is predominantly circulated in the form of ceruloplasmin, although a portion of it also exists in a free state. Copper is transported to various tissues and organs, particularly the liver, brain, heart, and skeletal muscle.

The liver serves as the primary storage site for copper in the body. Copper is stored in hepatocytes within the liver through the action of copper transport proteins such as ATP7B. Additionally, copper is stored in various tissues, such as the brain and heart.^11^ Copper plays a role in the functioning of various enzymes, including oxidases and superoxide dismutases, that are crucial for maintaining normal physiological functions in these tissues.

**FIGURE 1:**
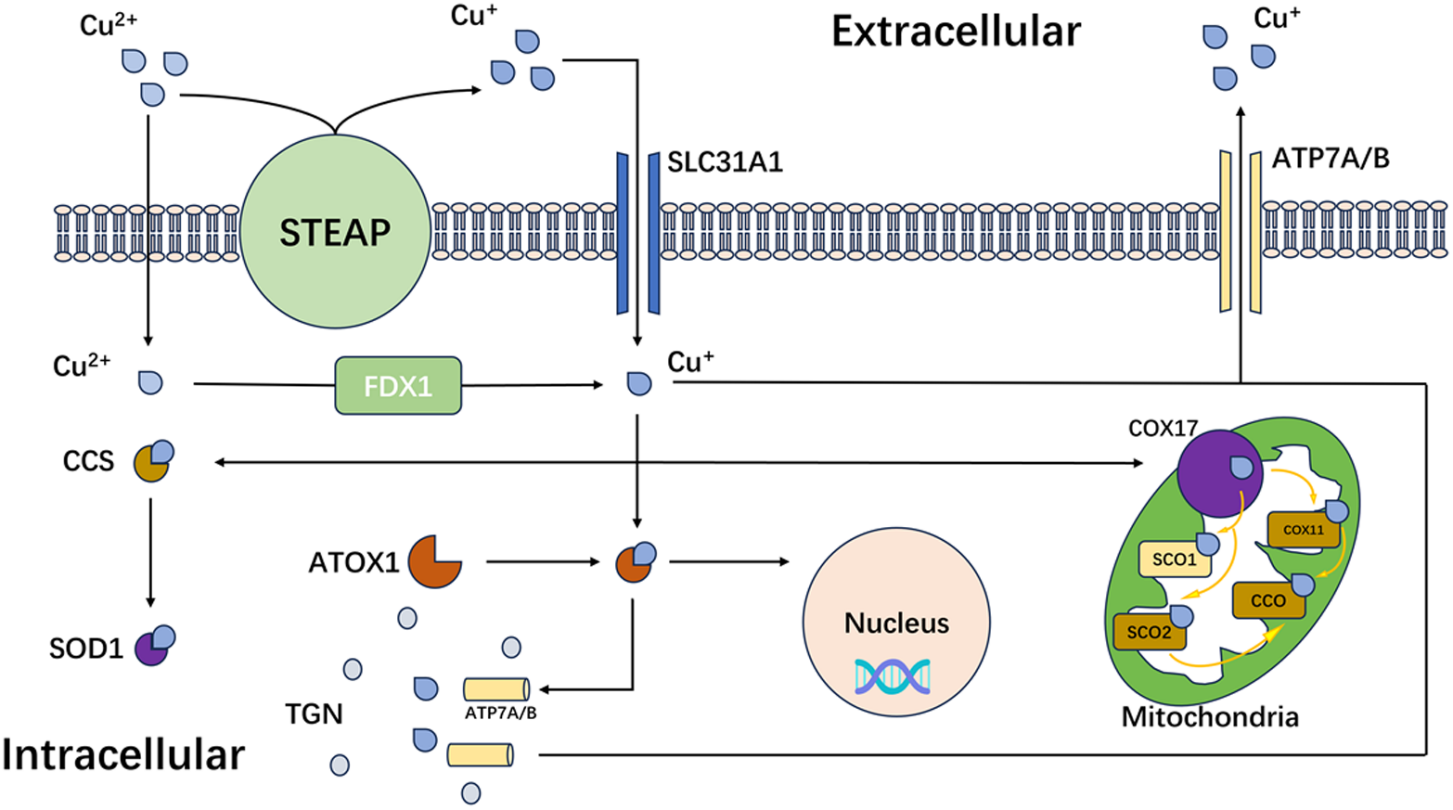
The metabolic pathway of copper within cells is a finely regulated process. Cu^2+^ from outside the cell can enter the cell through passive diffusion. Inside the cell, the reductase FDX1 can reduce Cu^2+^ to Cu^+^. Additionally, Cu^2+^ outside the cell can be reduced to Cu^+^ by the reductase STEAP and then transported into the cell with the assistance of CTR1. Within the cell, copper chaperones such as ATOX1, CCS, and SOD1 bind to copper ions and transport them to specific organelles to perform their biological functions. In the mitochondria, the protein COX17 transports copper ions from the cytoplasm to the mitochondrial inner membrane, where they are subsequently passed on to mitochondrial membrane proteins (SCO1 and SCO2) as well as COX11, ultimately combining with cytochrome c oxidase (CCO) to participate in the electron transport chain. In the cell's trans‐Golgi network (TGN), ATP7A and ATP7B are two important copper‐transporting ATPases that regulate the excretion of copper to maintain the homeostasis of intracellular copper ions. The coordinated action of these chaperones and transport proteins ensures the rational distribution and functional expression of copper ions within the cell while also preventing cytotoxicity that excess copper ions may cause.

Copper metabolism involves a complex network of enzymes and proteins in the body. Serving as an essential cofactor for numerous enzymes, copper plays a pivotal role in numerous biological processes, including iron metabolism, neurotransmitter synthesis, antioxidant defense, DNA repair, and energy production.^12^, ^13^ Copper metabolism also involves the regulation of its redox state, which is highly important for the control of intracellular signal transduction and gene expression.^14^ Copper is primarily excretion through bile. In the liver, excess copper is excreted into the intestine via bile, where some of it is removed from the body through feces, while another portion can be reabsorbed again in the intestine, forming a cycle^15^ (Figure 1). Moreover, copper can be excreted in small quantities through urine and sweat, although this results in only a minor loss of copper in the human body.

#### 2.1.2 Cellular regulation of copper

Copper, an essential trace element, is necessary for a wide range of physiological processes in nearly all cell types. Since the accumulation of copper within cells can cause oxidative stress and disrupt cellular function, the intake, distribution, and elimination of copper are strictly regulated. Copper homeostasis at the cellular level is coordinated by a series of copper‐dependent proteins, including copper enzymes, copper chaperones, and membrane transport proteins. These proteins work together to coordinate the import, export, and intracellular utilization of copper, thereby maintaining cellular copper levels within a specific range. This regulation helps to prevent the consequences of both copper excess and copper deficiency.

CTR1 is a transporter protein located on the cell membrane, primarily responsible for transporting copper ions (Cu^+^) from outside the cell to the inside.^16^ CTR1 consists of multiple regions containing methionine and histidine, which may endow it with high affinity and selectivity for copper ions and cisplatin, facilitating efficient Cu^+^ transport from the extracellular environment into the cell. In particular, a hydrophobic region at the N‐terminus, consisting of 60 amino acids, contains several such clusters, including the M2 region.^17^ Studies suggest that CTR1 may work in conjunction with some metal reductases (such as STEAP and DCYTB) to transport copper ions across the membrane.^18^, ^19^ The expression and activity of CTR1 are regulated by various factors, including intracellular copper demand, oxidative stress, and the activation of signaling pathways. For instance, when intracellular copper levels are low, the expression of CTR1 increases to enhance the uptake of copper ions; under oxidative stress conditions, the function of CTR1 may be affected, thereby impacting copper ion homeostasis.^20^ Kuo et al. found that mice experiencing copper deficiency exhibit enhanced CTR1 staining in the kidneys, duodenum, and choroid plexus when compared to mice with sufficient copper levels. This finding underscores the specialized role of CTR1 in the transport of copper ions.^21^, ^22^ Further recent studies indicate that cells respond to increased extracellular copper levels by upregulating the expression of Refrex1 expression, which regulates copper acquisition through interaction with the primary copper transporter CTR1.^23^ In yeast, CTR2 serves as a vacuolar copper export protein, capable of mobilizing copper from the vacuolar lumen to the cytoplasm when extracellular copper is scarce. In mammalian cells, CTR2 is believed to play a role in copper homeostasis, but the specific mechanism remains clear.^24^ Additionally, studies have shown that CTR2 physically interacts with CTR1 in vivo, potentially forming heterotrimeric or homotrimeric complexes of CTR1 and CTR2.^25^ Mice with complete knockout of both alleles of the CTR1 gene exhibit severe growth and developmental defects at mid‐gestation and die in utero, indicating that CTR1 is also crucial for normal embryonic development.^26^

In the cytoplasm, the transportation of copper is precisely regulated by a network of copper chaperones with high affinity and synergistic action. Antioxidant‐1 (ATOX1) plays a key role in the transfer of copper ions to ATP7A and ATP7B in the trans‐Golgi network (TGN), facilitating the biosynthesis of copper‐dependent enzymes such as lysyl oxidase, tyrosinase, and plasma copper blue protein 32.^27^ ATOX1 is a metallo‐chaperone protein that directly interacts with copper‐transporting ATPases and is crucial for perinatal copper homeostasis. Hamza et al. observed a significant increase in intracellular copper content in ATOX1‐deficient cells due to impaired copper excretion.^28^ These findings also reveal that ATOX1 is essential for establishing the threshold of copper‐dependent transport ATPases in the secretory pathway, and in the absence of ATOX1, this transport alone is not sufficient to restore normal copper excretion.^29^

SOD1 is an enzyme widely present in cells, responsible for eliminating superoxide and protecting cells from oxidative damage. The activity of SOD1 depends on its post‐translational modification, including the acquisition of copper and zinc, the formation of intramolecular disulfide bonds, and dimerization.^30^ CCS is responsible for the copper insertion and disulfide bond formation of SOD1 and regulates the localization of SOD1 between the cytoplasm and mitochondria. The transportation of SOD1 and CCS into the mitochondrial is regulated by the Mia40‐Erv1 disulfide relay system, which is associated with the function of the mitochondrial respiratory chain.^31^ Experiments have shown that the concentration of protein level of CCS increases during copper deficiency, and the degradation of CCS accelerates when cells transition from a state of copper deficiency to a state of copper sufficiency. Additionally, CCS may prioritize the utilization of copper by regulating its degradation when there is copper scarcity, which is crucial for maintaining the balance and functioning of intracellular copper.^32^

COX17 functions as a copper chaperone protein responsible for transporting copper ions from the cytoplasm to mitochondrial membrane proteins, specifically SCO1 and SCO2. These membrane proteins are responsible for incorporating copper into the mitochondrial‐encoded cytochrome c oxidase II (MT‐CO2/COX2). Additionally, COX17 can facilitate the transportation of copper from the cytoplasm to MT‐CO1/COX1 by delivering it to COX11.^3^, ^33^, ^34^ The active core of the cytochrome oxidase (COX) complex in humans consists of two key subunits, namely COX1 and COX2. These two subunits have a structural correspondence to the conserved CuB and CuA binding sites. These binding sites are specific locations where the subunits bind to copper ions, ensuring their normal function and efficiency in the mitochondrial electron transport chain.^35^ Studies have shown that the overexpression of COX17 in cells from patients with SCO2 mutations can partially restore COX activity. In addition, increasing copper concentration can also partially restore COX activity, further supporting the importance of COX17 in copper transfer and COX function.^36^ In summary, mutations in the COX17, SCO1, and SCO2 genes can disrupt the function of COX, affecting cellular respiration and potentially leading to the production of excessive reactive oxygen species (ROS), ultimately leading to cell death.^37^

Copper‐dependent ATPases, ATP7A and ATP7B, play complementary roles in regulating copper metabolism in the body. They utilize adenosine 5′‐triphosphate (ATP) to transport copper ions, thereby maintaining cellular and systemic copper balance. ATP7A plays a multifaceted function across various tissues, primarily transporting copper ions absorbed from the small intestine to cells throughout the body, except for the liver. In contrast, the primary function of ATP7B is the excretion of copper; it is active in the liver, where it regulates copper homeostasis by eliminating excess copper from the body through bile secretion to maintain a balance in copper metabolism.^38^ ATP7A and ATP7B exhibit dynamic movement within the cell, primarily shuttling between the endoplasmic reticulum (ER), the trans‐golgi network (TGN), and the cell membrane. This shuttling is facilitated by the endocytic recycling mechanism involving the retromer complex and other associated factors.^39^ The transport activity of ATP7A and ATP7B is dependent on the intracellular copper concentration. Under basal copper levels, these ATPases are located in the TGN; however, when copper concentrations rise, they are translocated to the cell periphery to remove excess copper ions.^40^, ^41^ The phosphorylation levels of ATP7A and ATP7B correspond to copper concentrations, which affects their intracellular distribution. It is widely accepted that hyperphosphorylation of ATP7A/B triggers their translocation from the TGN to the cell membrane, while dephosphorylation facilitates their retrieval.^42^ Recent studies suggest that the expression levels of Cu‐ATPases, particularly ATP7A and ATP7B, may be related to the occurrence and progression of cancer. For example, high expression of ATP7A in invasive breast cancer cells suggests that Cu‐ATPases may be involved in the metastasis process of cancer.^43^ Additionally, upregulation of ATP7A expression has been observed in pancreatic cancer cells^44^; however, whether this can directly impact the occurrence and progression of tumors remains unclear.

### 2.2 Molecular mechanisms of cuproptosis

#### 2.2.1 The role of copper ions in the TCA cycle

**FIGURE 2:**
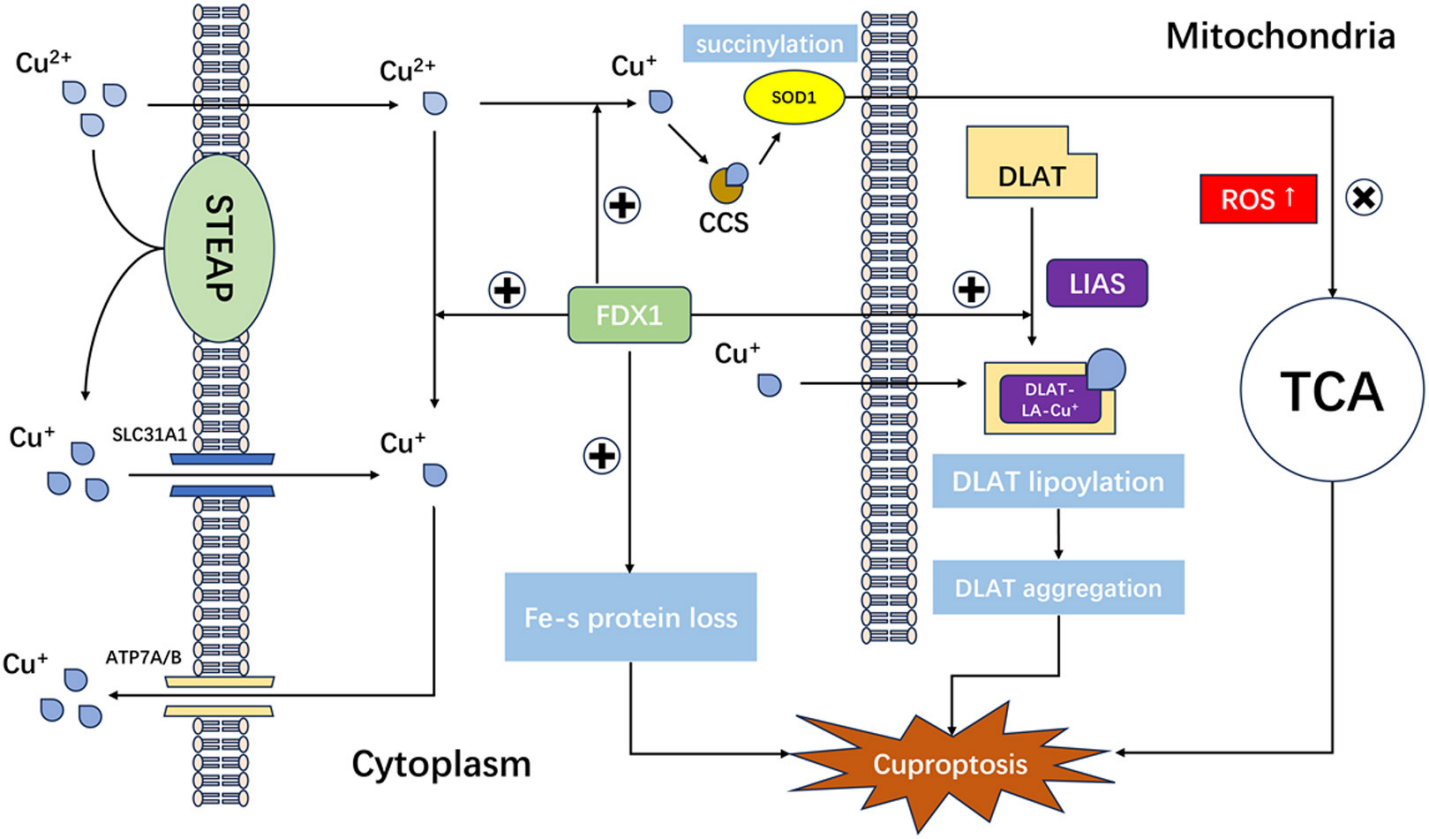
Cuproptosis is a complex process involving various molecular mechanisms. Initially, copper ions are absorbed into the cell through the mediation of the SLC31A1 protein. Subsequently, the ATP7A/B proteins transport copper ions from inside the cell to the outside, maintaining the homeostasis of copper ions. In this process, the FDX1 protein plays a crucial role. FDX1 reduces Cu^2+^ to Cu^+^, thereby promoting the process of palmitoylation of the DLAT protein. The binding of copper ions to palmitoylated proteins leads to their aggregation and loss of normal function, ultimately triggering cell death. Additionally, FDX1 is involved in the loss of iron–sulfur cluster proteins, which damages mitochondrial function and further induces cell death. Meanwhile, the binding of copper ions to the CCS protein triggers the succinylation of the SOD1 protein, which weakens the ability of SOD1 to clear reactive oxygen species (ROS). Excessive accumulation of ROS within the cell negatively affects the cell's tricarboxylic acid cycle (TCA cycle), ultimately leading to cell death.

Copper‐dependent death occurs through the direct binding of copper to acylated protein components in the TCA cycle^1^ (Figure 2). The TCA cycle is an important metabolic pathway within the cell, where some key enzymes require acylation modification to function. Acylation is a post‐translational modification that involves the covalent attachment of fatty acids or other lipid molecules to proteins, which can be either permanent or reversible.^45^ These acylated proteins include succinyl‐CoA transferase, glycine cleavage system protein H, succinyl‐CoA transferase, and succinyl‐CoA acetyltransferase, which regulate the carbon entry points of the TCA cycle.^46^ The interaction between copper and the TCA cycle involves the direct influence of copper on acylated proteins. The interaction between copper ions and acylated proteins, especially DLAT, is facilitated by the involvement of FDX1 (ferredoxin 1). FDX1 typically converts Cu^2+^ to Cu^+^, resulting in Cu^+^ binding more tightly and increased toxicity to acylated proteins.^47^ The binding of copper to acylated proteins can cause these proteins to aggregate, which may occur through copper bridging the thiol groups in the acylated proteins. The aggregation of acylated proteins may lead to the loss of their function and further induce protein toxicity stress within the cell.^48^ In addition, the binding and aggregation of copper with acylated proteins can lead to the loss of iron–sulfur cluster proteins, which can impair mitochondrial function, prevent the cell from maintaining normal metabolic activity, and thus affect cell survival.^49^ Copper‐induced aggregation of acylated proteins and the loss of iron–sulfur cluster proteins activate the cellular protein quality control mechanisms, such as the increased expression of heat shock protein 70 (HSP70), ultimately leading to cell death.^50^ In summary, cells that depend on mitochondrial respiration or have higher levels of acylated TCA enzymes are more sensitive to copper ionophore‐induced cell death.^51^

Moreover, related research has found that the binding of copper ionophore protein (CCS1) leads to the succinylation of cysteine residue (Cys146) on SOD1.^52^ As a crucial antioxidant enzyme, the succinylation of SOD1 may alter its catalytic function, thereby affecting its ability to eliminate superoxides. Consequently, this could increase superoxide levels, potentially impacting the activity of enzymes involved in redox reactions within the TCA cycle and induce cell death.^53^, ^54^ However, current research on the specific mechanisms and extent of this impact is relatively limited. Future studies may provide more detailed information on how succinylation of SOD1 regulates the TCA cycle and the implications of this regulation on cellular metabolism and the progression of diseases.

#### 2.2.2 Redox activity of copper ions

The interaction between copper ions and hydrogen peroxide can result in the production of ROS, a process also known as the Fenton reaction. However, the catalytic efficiency of copper ions is generally lower than that of iron ions.^55^, ^56^ At low concentrations of iron ions, the presence of copper ions can significantly promote the generation of ROS. In the context of copper‐induced cell damage, the production of ROS and its impact is multifaceted; ROS can directly damage cellular components and also indirectly lead to cell damage by affecting cellular signaling and gene expression.^57^, ^58^ Furthermore, recent studies have found that copper ions can enhance oxidative stress and endoplasmic reticulum stress responses.^59^ This can exacerbate zinc ion‐induced neuronal cell death by activating the SAPK/JNK signaling pathway,^60^ which may be one of the key mechanisms underlying cuproptosis.

#### 2.2.3 Copper ions and DNA damage

The programmed cell death induced by copper may also be associated with its interaction with DNA. Research by Anchordoquy et al. reported that culturing bovine cumulus cells in an environment with high concentrations of copper significantly increased their genetic damage index (GDI), and an increase in DNA damage was observed across all tested concentrations of copper.^61^ These findings suggest that copper may induce the death of bovine cumulus cells by producing genotoxic effects. Additionally, other studies have found that nanoparticles of CuO exhibit stronger genotoxic activity than microparticles of CuO when administered at the same dosage.^62^, ^63^ This is attributed to nanoparticles' higher cellular uptake, leading to an increased level of copper in the cells. Although the induction of cell death by copper through the initiation of DNA damage has been demonstrated in numerous experiments,^64^, ^65^, ^66^ it remains unclear whether copper can directly interact with DNA. Current research indicates that redox‐active metal ions interact with the bases found in DNA molecules, which may lead to DNA strand breaks and alterations in the chemical structure of the bases.^67^ Therefore, the presence of copper ions may promote abnormal base pairing through oxidative modification of the bases, resulting in base substitution mutations. Understanding the mechanism by which copper ions induce base substitution mutations is of significant importance and may represent a unique direction for research into cuproptosis.

#### 2.2.4 Cuproptosis and apoptosis

Apoptosis is a programmed cell death process mediated by both intrinsic and extrinsic signals.^68^ The intrinsic pathway, also known as the mitochondrial pathway, primarily responds to internal cellular stress signals, such as DNA damage or the absence of growth factors, which can activate a series of protein kinases, including the p53 protein.^69^ The activation of the p53 protein can further promote the expression of proteins, such as Bax and Bak, which play a key role in regulating mitochondrial membrane permeability.^70^ The activation of Bax and Bak leads to increased mitochondrial outer membrane permeability, facilitating the release of cytochrome c.^71^ Cytochrome c, once released, binds with apoptotic protease activating factor 1 (APAF‐1) and the precursor of caspase‐9 (procaspase‐9) to form the apoptosome.^72^ The apoptosome further activates caspase‐9, which subsequently activates downstream effector caspases, such as caspase‐3, ‐6, and ‐7.^73^ These effector caspases are responsible for executing the programmed cell death.

Cuproptosis is a newly discovered form of cell death that involves the interaction of copper ions with proteins associated with the mitochondrial respiratory chain. However, it has been noted that simply inhibiting apoptosis is insufficient to prevent elesclomol‐induced cell death. Conversely, the use of copper chelators to treat cell death caused by an excess of Cu^2+^ can demonstrate a significant protective effect. This indicates that the mechanism of cell death caused by copper toxicity is inherently different from that of apoptosis.^1^ In addition, copper‐induced neurodegeneration and oxidative damage can promote the expression of apoptosis‐related proteins Bax, copper‐induced cell death‐related proteins FDX1 and DLAT, and the protein toxicity stress marker HSP70, while reducing the expression of Fe‐S cluster proteins.^74^ Further research has shown that an excess Cu can lead to the accumulation of ROS within cells and a significant increase in levels of SOD and LPO. Concurrently, this state of copper excess can upregulate the mRNA and protein expression levels of Bak1, Bax, CytC, and Caspase3, while suppressing the mRNA and protein expression of Bcl2, exhibiting a clear dose‐dependent effect, thereby inducing apoptosis.^75^ These findings reveal a potential intrinsic connection between copper‐induced cell death and apoptosis. Although the two processes differ in molecular mechanisms, they may influence each other in the regulation of programmed cell death.

#### 2.2.5 Copper and autophagy

Autophagy is an intracellular process characterized by the formation of double‐membrane structures that facilitate self‐digestion.^76^ It plays a crucial role in clearing damaged or misfolded proteins that could potentially lead to disease, thereby maintaining cellular homeostasis and health.^77^ The initiation of autophagy is regulated by multiple signaling pathways, including the AMPK‐mTOR pathway. The mTOR kinase serves as a pivotal regulator of autophagy; its activation suppresses autophagy, while its inhibition promotes it. AMPK enhances autophagy by phosphorylating the mTOR complex 1 (mTORC1),^78^ whereas active mTORC1 inhibits autophagy by binding to and phosphorylating the ULK1/2 (unc‐51 like autophagy activating kinase 1/2) and ATG13 (autophagy‐related 13) protein complex.^79^ The ULK1 complex is an essential positive regulator for autophagosome formation, which recruits the PI3K complex to function collaboratively in this process.^80^ The fusion of autophagosomes with lysosomes requires the participation of LAMP‐1 and Rab7 proteins, followed by the degradation of the contents within the autophagosomes.^81^ Copper, through its redox activity, can induce autophagy by generating ROS via the Fenton reaction. Excessive ROS can hyperactivate the autophagic mechanism, providing positive feedback that accelerates cell death by selectively degrading damage‐resistant or antioxidant proteins.^82^ It has been reported that copper can modulate the AMPK‐MTOR pathway by increasing ATG expression, inducing oxidative stress through the mitochondrial pathway, thereby triggering autophagy.^83^ The knockout of the cellular ATP7B leads to a significant increase in the expression of autophagy genes. This occurs as reduced mTOR activity results in the translocation of the mTOR substrate transcription factor EB to the nucleus, which then triggers the activation of autophagy‐related genes.^84^ Copper can also induce autophagic flux by directly binding and activating the ULK1/2 in tumor cells. In addition, the absence of Ctr1 or mutations in ULK1 results in reduced Cu binding and impaired formation of the autophagosome complex.^85^ Recent reports indicate that copper promotes the autophagic degradation of GPX4 protein, mediated by TAX1BP1, which can facilitate ferroptosis in pancreatic cancer cells.^86^ However, the role of copper in cellular autophagy is dualistic; the use of copper chelators or the knockout of SLC31A1 increases autophagy in pancreatic cancer cells.^87^ Further in‐depth research into the mechanisms by which copper affects cellular autophagy and the evaluation of its clinical application prospects are necessary. Such research will be instrumental in providing insights for future cancer therapies.

### 2.3 Copper‐dependent death and disease

Imbalances in copper metabolism can lead to various adverse effects on human health. These harmful effects can result from disturbances in copper absorption, reduced copper excretion, and abnormal accumulation of copper in different vital organs. Additionally, studies have shown that dysregulation of copper homeostasis is closely related to the occurrence and progression of certain cancers.

#### 2.3.1 Diseases caused by copper deficiency

Menkes disease (MD), a representative of copper metabolism disorders, is an X‐linked lethal neurodegenerative condition caused by mutations in the ATP7A gene, which encodes a copper‐transporting P1B‐type ATPase.^88^ Mutations in the ATP7A gene lead to impaired function of the ATP7A protein, preventing the transport of copper from intestinal cells to other vital organs, and resulting in systemic copper deficiency.^89^ Consequently, copper cannot be effectively delivered to copper‐dependent enzymes, such as cytochrome c oxidase and superoxide dismutase, leading to a decrease in their activity and adverse effects on key physiological processes such as cellular respiration, neurotransmitter synthesis, free radical scavenging, and melanin production.^90^, ^91^ MD is a highly lethal disease, with typical clinical manifestations in the neonatal period, including hypothermia, hypoglycemia, malnutrition, and occasionally hemorrhagic damage, such as cephalohematoma.^92^, ^93^ Older infants often exhibit seizures and neurodegenerative changes, likely due to reduced activity of dopamine‐β‐hydroxylase (DβH),^90^ impaired mitochondrial function from reduced COX activity, and cerebral lactic acidosis caused by mitochondrial dysfunction. In addition to the clinical manifestations caused by copper deficiency in the brain tissue, connective tissue dysfunction is also a classic feature of MD, most notably characterized by colorless and brittle hair.^93^ This is likely due to the weakened function of ATP7A, inhibiting the activity of lipoxygenase (LOX) in the skin, muscles, and connective tissues, thereby hindering the normal oxidation of lysine and hydroxylysine within elastin and collagen.^94^ Reduced activity of LOX, decreases the formation of covalent cross‐links, which is essential for maintaining the tensile strength and elasticity of connective tissues in the skeleton, muscles, and cardiovascular system.^95^, ^96^

Genetic screening is usually the most effective diagnostic method for MD,^97^ and the analysis of plasma catecholamine levels can also accurately diagnose neonatal MD.^98^ Because the essence of the disease is the inability to transport copper from intestinal cells to other tissues and organs, direct copper supplementation may be an effective treatment method for MD. Currently, the use of copper salts (such as copper‐histidine complex) that can be directly transported through the bloodstream is the primary clinical treatment for MD.^99^ This treatment is effective in patients with ATP7A mutations that still allow copper to cross the blood–brain barrier.^100^ In addition, patients diagnosed prenatally or shortly after birth and receiving early treatment have longer survival time and better prognoses,^101^ which suggests that copper is crucial for the development of the human nervous and motor systems. Interestingly, patients with asymptomatic MD respond better to copper‐histidine complex treatment compared to those with typical symptoms,^102^ which may be related to the duration and extent of their copper deficiency.

In addition to the classic MD, studies have found that copper deficiency may also be associated with anemia, specifically copper‐deficiency anemia. Various causes can lead to copper deficiency in the human body, such as zinc‐induced copper deficiency, copper loss due to surgery, copper deficiency in parenteral or enteral nutrition owing to celiac disease, etc.^103^, ^104^, ^105^ Copper deficiency can impair the activity of iron oxidases and disrupt the transportation of iron from cells to plasma, thereby affecting hemoglobin synthesis.^106^, ^107^ However, whether copper deficiency directly impacts the hematopoietic function of the bone marrow is currently unknown. Measuring serum copper, serum ceruloplasmin, or 24‐hour urinary copper levels in conjunction with bone marrow biopsy is important for diagnosing copper‐deficiency anemia.^108^ Additionally, there are case reports suggesting that copper deficiency may cause spinal cord lesions.^109^ Although the importance of copper to the nervous system has been confirmed, the pathogenesis of copper‐deficiency myelopathy remains to be explored.

#### 2.3.2 Diseases caused by copper overload

Just as abnormal copper accumulation within cells can induce cell death, copper overload in the human body can also lead to the onset of related diseases, the most notable being Wilson's disease (WD). WD is an autosomal recessive genetic disorder caused by mutations in the ATP7B gene, which encodes a transmembrane copper‐transporting ATPase primarily responsible for transporting copper out of cells.^110^ The loss of ATP7B function leads to abnormal accumulation of copper in vital organs and tissues in the human body, such as the liver and brain, thereby causing a range of clinical symptoms.^111^

Copper is primarily excreted from the body through bile, making the liver the main regulatory organ for copper homeostasis. Consequently, liver pathology is often the first and most common manifestation of WD. The hallmark of this condition is an abnormally elevated copper concentration in hepatocytes of patients with WD, which initially bind to metallothionein in the cell cytoplasm. As copper accumulation intensifies, copper ions are released from their bound state with metallothionein and may transfer to other parts of the cell, especially lysosomes.^112^ Within lysosomes, excessive copper can disrupt their normal degradation function and damage cellular structures, including proteins, lipids, DNA, and RNA, through the generation of oxidative stress.^113^

In addition, the accumulation of copper can trigger apoptosis pathways, leading to hepatocyte death, through the activation of acid sphingomyelinase and other pathways.^114^ Long‐term copper accumulation and cell death can lead to a series of pathological changes in the liver, such as fatty liver, hepatitis, and hepatic fibrosis, potentially progressing to cirrhosis. Mutations in the ATP7B gene disrupt the transport function of ATP7B and impair the synthesis of ceruloplasmin, an important copper‐binding protein.^115^ As hepatocytes perish and plasma ceruloplasmin levels decline, the concentration of free copper ions in the bloodstream increases, facilitating the transportation of copper to various tissues and organs via the blood. Once copper accumulation reaches a critical threshold, it induces clinical symptoms, with brain dysfunction being the most significant, followed by effects on the eyes, heart, bones, and other organs.^111^, ^116^

Moreover, relevant studies have confirmed a positive correlation between copper concentration in brain tissue cells and neurological damage.^117^ Astrocytes, a type of glial cell in the central nervous system, play multiple important roles in the brain. These roles include supporting and protecting neurons, maintaining the stability of the extracellular environment, forming the blood–brain barrier, and participating in the repair process of the nervous system.^118^ Astrocytes can take up excess copper, upregulate metallothionein levels, and mitigate copper's neurotoxicity by increasing cell number and volume.^117^ However, long‐term exposure to a high copper concentration may damage and eventually kill astrocytes, further impairing the blood–brain barrier and causing damage to neurons and other brain tissues.^119^ The clinical manifestations of brain damage in patients with WD vary according to the affected cerebral region, with the most common and severe manifestation being damage to the putamen, clinically presenting as dystonia and Parkinson's disease.^120^

Another important diagnostic feature observed in patients with WD is the Kayser–Fleischer ring, resulting from abnormal copper accumulation in the eyes, which is especially common among patients with neurological damage.^121^ While measuring serum ceruloplasmin levels and nonceruloplasmin‐bound copper levels can serve as an effective method for diagnosing WD, they often lack specificity.^122^, ^123^, ^124^ In addition, clinical manifestations and imaging examinations serve as supplementary diagnostic tools for WD, while DNA analysis of ATP7B gene mutations provides genetic confirmation. WD is a systemic disease caused by mutations in the ATP7B gene, which lead to abnormal copper accumulation. Treatment primarily involves regulating copper intake and enhancing copper excretion, which can be maintained for life.^121^, ^125^ Moreover, liver transplantation is considered an effective treatment for WD due to its primary manifestation as liver damage; however, there are still several limitations in its therapeutic effects.^126^ Furthermore, owing to the role of astrocytes in copper metabolism and neuroprotection, they could be potential targets for WD treatment strategies. These strategies may involve enhancing their copper buffering capacity or antioxidant defenses to reduce the toxicity of copper.

#### 2.3.3 Copper and cancer

Copper, an essential trace element for the human body, exhibits a dual effect on cells due to its redox properties. Copper influences cellular signal transduction by activating or inhibiting specific copper‐dependent enzymes. For instance, in the RAF–MEK–ERK signaling pathway, copper can enhance the kinase activity of MEK1 and MEK2 through metal isoform regulation, leading to the phosphorylation of ERK1 and ERK2, thereby regulating the expression of genes associated with cell proliferation.^127^ Several reports indicate that the plasma and cellular copper concentrations in patients with cancer are higher than normal levels.^128^, ^129^, ^130^, ^131^, ^132^, ^133^, ^134^ Simultaneously, copper can directly activate various pro‐angiogenic factors (such as VEGF, FGF2, etc.) to promote new blood vessel formation, a key step in tumor growth and metastasis.^135^, ^136^ The role of copper in cell biology extends beyond maintaining basic metabolic processes; it also participates in more complex cellular behaviors. For example, it acts on the ATOX‐ATP7A‐LOX signaling pathway, where ATOX1, as a cytosolic copper chaperone, transfer copper to ATP7A, thereby regulating the intracellular distribution and export. The LOX family of enzymes uses copper as a cofactor to catalyze the cross‐linking of collagen and elastin, affecting the structure and function of the extracellular matrix, which can promote the invasive expansion of tumors.^137^ This demonstrates the relationship between copper and the onset and development of cancer, with tumor tissues requiring more copper compared to healthy tissues.^138^ Considering copper's role in cancer development, it emerges as a potential target for therapeutic intervention. Current potential cancer treatment strategies primarily involve two approaches: using chelating agents to counteract excess copper and increasing copper concentration to mediate programmed cell death in tumor cells.^139^ Copper chelating agents such as triethylenethiophosphoramide (TTM) or D‐penicillamine can form stable complexes with copper ions in the body, thereby reducing the concentration of available copper ions within cells and inhibiting the activity of angiogenic factors.^140^, ^141^ Protein aggregation induced by copper ions and the loss of Fe‐S cluster proteins trigger a protein toxicity stress response, activating the cell's unfolded protein response (UPR) and autophagy process. However, when these mechanisms fail to clear misfolded proteins, they lead to cell death.^142^

Copper ionophores (such as elesclomol, DSF, etc.) primarily enhance intracellular copper levels, resulting in mitochondrial dysfunction and the production of ROS. These combined effects ultimately promote copper‐induced death, contributing to their anti‐tumor effects.^143^, ^144^, ^145^ Several studies have found that the concurrent administration of DSF and copper treatment can downregulate the expression of tumor suppressor gene PTEN and activate the AKT signaling pathway.^145^ This lays a theoretical basis for future research into the synergistic use of copper ionophores with PI3K‐AKT pathway inhibitors. In addition, due to DSF's effective penetration of blood–brain barrier, relevant studies have confirmed that its combination treatment with temozolomide can significantly extend the survival period of patients with specific types of cancer.^146^

## 3 CONCLUSION

Copper, as one of the essential trace elements for the human body, plays an irreplaceable role in both the macro and micro regulatory processes. It serves as an indispensable cofactor for certain key enzymes, and a deficiency in copper can lead to a series of adverse effects on the organism. However, an excess of copper can induce oxidative stress and trigger cell death through its action on the mitochondrial respiratory chain, a process known as cuproptosis. As a novel form of cell death, cuproptosis provides a new perspective on the role of copper ions in cellular respiration metabolism and diseases. While the acylation of proteins in the mitochondrial respiratory chain and the lipoic acid pathway have been identified as crucial in cuproptosis, exploring alternative pathways through which copper may induce cell death is a significant research avenue. In addition, there are no detailed reports on how the acylated mitochondrial respiratory chain proteins trigger cell death through specific signaling pathways. Therefore, further research is needed to clarify the exact mechanism of cuproptosis and to address some intractable diseases in clinical practice through this process. Incidentally, although the biomarkers for copper poisoning are not yet clear, identifying their specific markers is of great significance for promoting the clinical diagnosis and treatment of copper poisoning under human pathological conditions, which will be a key direction for future research.

Programmed cell death induced by metal ions has gradually gained significant attention in recent years. One example is ferroptosis, a form of cell death induced by iron ions causing lipid peroxidation of the cell membrane, leading to an excessive accumulation of ROS and ultimately triggering programmed necrosis. Interestingly, mitochondria have emerged as a key factor in both processes, as they are responsible for cell deaths induced by metal ions. This suggests the potential for interaction between the two. Recent studies have confirmed that mitochondrial glutathione (GSH) can effectively downregulate Cu‐induced cell death by inhibiting enzyme acylation and promoting DLAT oligomerization. Additionally, in the management of ferroptosis, the use of MitoQ as a ROS scavenger can increase GSH levels and maintain the integrity of mitochondrial. During ferroptosis, mitochondria undergo a series of morphological changes; however, it remains unclear whether mitochondria undergo similar morphological changes in mitochondria during cuproptosis. Exploring the relationship between iron and copper, the two metal ions in cell death may emerge as a promising research direction.

By analyzing the various diseases resulting from the disturbance of copper homeostasis in the body, a significant correlation between these diseases and cuproptosis can be identified. Therefore, while investigating cuproptosis, additional therapeutic targets for diseases characterized by copper ion disorders can be uncovered. To better understand the dynamic process through which copper toxicity triggers and accelerates the advancement of diseases, further evidence is required. In the future, prospective studies will be particularly crucial in conditions such as WD, and neurodegenerative disorders, notably in oncology, where the induction and regulation of cuproptosis could yield great therapeutic potential. This will involve exploring different copper ion carriers and the precise regulation of copper ion levels across various tissues.

In summary, the field of cuproptosis research is rapidly expanding. Therefore, future research should explore the molecular mechanisms of cuproptosis, develop new therapeutic strategies, and verify their safety and efficacy in clinical trials.

## AUTHOR CONTRIBUTIONS

Chengliang Pan was a major contributor in writing the manuscript. Pengfei Ge guided the direction of the writing for this article. Other authors assisted in completing the literature search and organization‐related tasks. All authors read and approved the final manuscript.

## FUNDING INFORMATION

This work is funded by the National Natural Science Foundation of China (82372690, 82173027).

## CONFLICT OF INTEREST STATEMENT

The authors declare that they have no competing interests.

## Data Availability

Data sharing is not applicable to this article as no new data were created or analyzed in this study.
